# Decrease of invasive pneumococcal disease (IPD) in adults after introduction of pneumococcal 13-valent conjugate vaccine in Spain

**DOI:** 10.1371/journal.pone.0175224

**Published:** 2017-04-06

**Authors:** Jordi Càmara, José María Marimón, Emilia Cercenado, Nieves Larrosa, María Dolores Quesada, Dionísia Fontanals, Meritxell Cubero, Emilio Pérez-Trallero, Asunción Fenoll, Josefina Liñares, Carmen Ardanuy

**Affiliations:** 1 Hosp. Univ. de Bellvitge-Universitat de Barcelona-IDIBELL, L'Hospitalet de Llobregat, Spain; 2 Hosp. Univ. Donostia, Donostia-San Sebastián, Spain; 3 CIBER Enfermedades Respiratorias, Madrid, Spain; 4 Hosp. General Univ. Gregorio Marañón, Madrid, Spain; 5 Hosp. Univ. Vall d'Hebrón, Barcelona, Spain; 6 H. U. Germans Trias i Pujol, Badalona, Spain; 7 Corp. Sanitària Parc Taulí, IU-UAB, Sabadell, Spain; 8 Laboratorio de Referencia de neumococo, ISCIII, Madrid, Spain; Instituto Butantan, BRAZIL

## Abstract

A prospective laboratory-based multicenter study that collected all adult invasive pneumococcal disease (IPD) episodes from 6 Spanish hospitals before (2008–2009) and after (2012–2013). The 13-valent pneumococcal conjugate vaccine (PCV13) licensure was conducted in order to analyze the impact of PCV13 introduction for children on adult IPD. A total of 1558 IPD episodes were detected. The incidence of IPD decreased significantly in the second period by -33.9% (95% CI, -40.3% to -26.8%). IPD due to PCV7 serotypes (-52.7%; 95% CI, -64.2% to -37.5%) and to PCV13 additional serotypes (-55.0% 95% CI, -62.0% to -46.7%) significantly decreased whereas IPD due to non-PCV13 serotypes remained stable (1.0% 95% CI, -12.9% to 17.2%). IPD due to all PCV13 additional serotypes significantly declined with the exception of serotype 3 (-11.3%; 95%CI -35.0% to 21.1%). IPD due to two non-PCV13 serotypes varied: serotype 6C that rose (301.6%; 95%CI, 92.7% to 733.3%, p<0.001), related to the expansion of ST386^6C^, and serotype 8 that decreased (-34.9%, 95%CI, -57.1 to -1.2, p = 0.049), related to a decline of the ST63^8^. The recombinant clone ST6521^11A^ (variant of ST156^9V^) increased in frequency. The decrease of serotype 19A IPD was linked to a fall in those antibiotic susceptible clones. In the last period, rates of penicillin- and cefotaxime-resistance remained under 10% and 4%, respectively. Adult IPD decreased after the PCV13 introduction in Spain due to herd protection. The spread of multidrug resistant clones (ST386^6C^, ST6521^11A^) related to non-PCV13 serotypes needs further surveillance.

## Introduction

*Streptococcus pneumoniae* is a leading cause of severe disease worldwide, mainly affecting children and the elderly populations. Pneumococcus infection can cause a broad spectrum of invasive disease including bacteremic pneumonia, sepsis and meningitis. In 2015, it was estimated that pneumoccal pneumonia was responsible for more than 1.5 million deaths worldwide [1].

Based on the seven most frequent pediatric serotypes in the 1990’s (4, 6B, 9V, 14, 18C, 19F, and 23F), the heptavalent conjugate vaccine (PCV7) was developed and introduced in 2000–2001. Thereafter, the incidence of invasive pneumococcal disease (IPD) dramatically decreased among children due to PCV7-serotypes, but also among adults due to herd protection [2,3]. This change was accompanied by a general decline in antibiotic resistance [3,4]. However, new emerging serotypes were detected in the late PCV7 period [5–7] and the vaccine was improved by including six additional serotypes (PCV13: 1, 3, 5, 6A, 7F, and 19A), whose frequency increased after the PCV7 introduction. This PCV13 was licensed in Spain in 2010 for children and in 2012 for adults. Children’s vaccination occurs mainly in the private market, with the exception of the Autonomous Community of Madrid, which included universal PCV7 vaccination for children in 2009 (3+1 schedule) that was replaced by PCV13 in June 2010 with a coverage higher than 94% [8].

The purpose of our study was to evaluate the impact of the PCV13 introduction on the incidence of adult IPD in Spain and its relationship with the dynamics of genotypes and serotypes. To assess it, we designed a prospective multicenter study involving six Spanish teaching hospitals from three Spanish regions that serve a population of approximately 4.000.000 adult inhabitants. The study was conducted throughout two periods: prePCV13 (2008–2009) and PCV13 (2012–2013).

## Material and methods

A 4 year laboratory-based multicenter study involving 6 Spanish hospitals was conducted. The included hospitals serve a global population of around 4.000.000 adult inhabitants from three Spanish regions: Catalonia (Hospital Universitari de Bellvitge, Hospital Universitari Vall d’Hebron, Hospital Universitari Germans Trias i Pujol and Corporació Sanitària Parc Taulí), Madrid (Hospital General Universitario Gregorio Marañón) and the Basque Country (Hospital Universitario Donostia). An IPD episode was defined as the isolation of pneumococci from a normally sterile body site in a patient with clinical symptoms of infection (http://wwwn.cdc.gov/nndss/conditions/invasive-pneumococcal-disease/case-definition/2010/). All episodes of IPD among adults (≥18 years old) were prospectively collected. Only one isolate per episode was included. Data collection included age, sex, source of isolate and focus of infection. For comparison purposes, two periods were defined: pre-PCV13 (2008–2009) and PCV13 (2012–2013). The PCV7 (targeting serotypes 4, 6B, 9V, 14, 18C, 19F and 23F) was licensed in 2001 and the PCV13 in 2010 (targeting PCV7 serotypes plus 1, 3, 5, 6A, 7F and 19A). The incidence of IPD was calculated using the total number of people as the denominator which was obtained from data published by the regional governments (http://www.madrid.org/iestadis/, http://www.idescat.cat/es/ and www.eustat.eus/). The distribution of IPD episodes (using previous description) with missing serotype was assumed to be identical to those episodes with serotype information (45 episodes in the pre-PCV13 period and 10 in the PCV13 period).

This study was approved by the Clinical Research Ethics Committee of Hospital Universitari de Bellvitge. Written informed consent was considered not necessary. Patients’ data were anonymized for the purposes of this analysis.

### Bacterial strains, serotyping and antimicrobial susceptibility

All available isolates [904 of 949 (95.3%) from the pre-PCV13 period and 599 of 609 (98.4%) from the PCV13 period] were serotyped by Quellung reaction at the Spanish Reference Laboratory. Susceptibility to 8 antimicrobials (penicillin, cefotaxime, erythromycin, clindamycin, chloramphenicol, tetracycline, co-trimoxazole and levofloxacin) was tested by microdilution method following The Clinical and Laboratory Standards Institute (CLSI) recommendations and criteria [9]. For the epidemiological analysis of penicillin resistance, the oral therapy breakpoints were applied. A multidrug resistant (MDR) isolate was defined as non-susceptible to penicillin (MIC≥0.12) plus resistant to ≥2 classes of non-βlactam antimicrobials [10].

### Molecular typing

A selection of isolates was genotyped through PFGE and/or MLST. We analysed 378 isolates obtained in the pre-PCV13 period (39.8%) including most penicillin- or macrolide- resistant isolates (n = 162) and a selection of susceptible isolates (n = 206). We also analysed 474 isolates collected in the PCV13 period (77.8%). The stains were selected according to major serotypes and were representative of all the regions. All 852 isolates were typed by PFGE (*SmaI*). Band patterns were visually compared following the criteria described by Tenover [11,12]. Major PFGE patterns were described as those accounting for at least 5 isolates. In addition, 344 isolates (22.1%) were typed using MLST (Multi Locus Sequence Type) [13] including at least one representative isolate of each serotype-PFGE pattern combination.

### Statistical analysis

Statistical analysis was performed using the SPSS software package (SPSS, version 14.0; SPSS, Chicago, Illinois, USA). Statistical differences were assessed using the x2 or Fisher’s exact test when appropriate. Statistical significance was established at α = 0.05. All reported p values are two tailed. Incidence rates of IPD were defined as the number of episodes per 100.000 population and 95% confidence intervals (CIs) were calculated.

## Results

### Study population

A total of 1558 IPD episodes were detected. Of these, 949 (60.9%) were collected in the pre-PCV13 period and 609 (39.0%) in the PCV13 period. The overall mean age of patients was 61.6 years (range 18–107) and 925 (59.4%) episodes were detected in men. 839 episodes (53.9%) occurred in young adults (18–64 years), of these 458 (54.6%) were aged below 50. On the other hand, 719 episodes (46.1%) occurred in older adults (≥65 years), of these 434 (60.4%) were aged 75 or over. Demographic characteristics of the study population are shown in Table 1. No differences were found between the two periods in male percentage or source of isolates. The average age of the patients increased significantly during the study period (59.5 vs 64.7 years, p<0.001). The incidence of bacteremic pneumonia statistically decreased (9.03 vs 6.00 per 100 000 population, p<0.001) whereas the incidence of meningitis remained stable (1.03 vs 0.87 per 100 000 population, p = 0.33).

**Table 1:** Demographic characteristics, source of isolates and focus of infection of the study population.

		pre-PCV13 (2008–2009)		PCV13 (2012–2013)		p*
**Demographics**	**Age mean (years)**	59.5		64.7		<0.001
	**Male (%)**	60.0		58.5		0.56
**Source of isolates**		**Episodes(n)**	**%**	**Episodes(n)**	**%**	
	**Blood culture**	822	86.6	520	85.4	0.43
	**Cerebrospinal fluid**	47	5.0	41	6.7	0.14
	**Pleural fluid**	54	5.7	28	4.6	0.35
	**Ascitic fluid**	15	1.6	13	2.1	0.42
	**Other**	11	1.2	7	1.1	0.99
**Focus of infection**		**Episodes(n)**	Incidence**	**Episodes(n)**	Incidence**	
	**Pneumonia**	700	9.03	451	6.00	<0.001
	**Meningitis**	80	1.03	66	0.87	0.33
	**Peritonitis**	33	0.43	23	0.31	0.22
	**Unknown focus**	102	1.31	48	0.64	<0.001
	**Other**	34	0.44	21	0.28	0.10

* p-value comparing pre-PCV13 and PCV13 periods

** Number of episodes per 100.000 population.

### Incidence of invasive pneumococcal disease (IPD)

**Fig 1:**
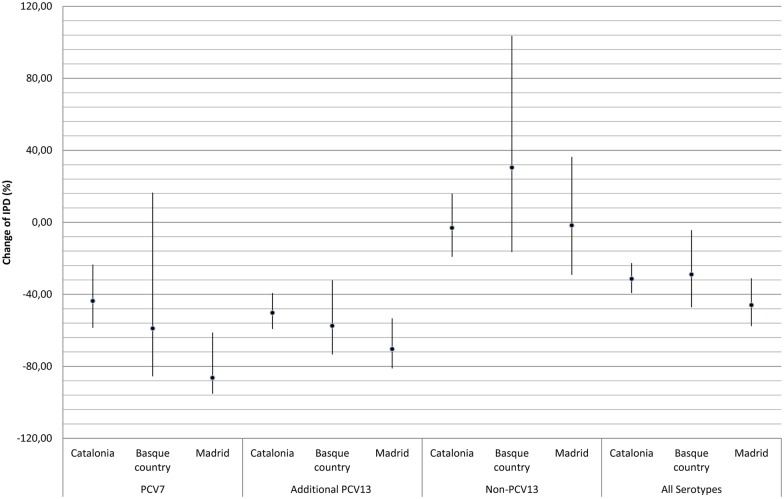
Regional changes in IPD by serotype group. Dots expressed the percentage of change and lines the limits of 95% CI. The decrease of IPD due to PCV7 and PCV13 additional serotypes was more noticeable in Madrid (the only region that included universal children vaccination with PCV13) than in the Basque Country or Catalonia.

The overall incidence of IPD decreased by -33.9% (95% CI, -40.3% to -26.8%) from 12.3 to 8.1 episodes per 100,000 population (Table 2). This decrease was statistically significant for all age groups. IPD due to PCV7 serotypes decreased 2.0 to 1.0 episodes per 100,000 (-52.7%; 95% CI, -64.2% to -37.5%) and IPD due to PCV13 additional serotypes declined from 5.7 to 2.6 episodes per 100,000 (-55.0%; 95% CI, -46.7% to -62.0%). The incidence of IPD due to non-PCV13 serotypes remained stable: 1.0% (95%CI, -12.9% to 17.2%), from 4.5 to 4.6 episodes per 100 000 population. Among patients aged 18–50 years, the incidence of IPD due to both PCV13 and non-PCV13 serotypes showed a statistically significant decrease. In those groups of patients aged >50 years, the IPD incidence due to PCV13 additional serotypes decreased (p<0.001) whereas the IPD incidence due to non-PCV13 serotypes increased, although this difference was not statistically significant. Since the hospitals are located in three different Spanish regions, we also analyzed IPD changes by regions. The decrease of IPD due to PCV7 and PCV13 additional serotypes was more noticeable in Madrid than in the Basque Country or Catalonia, although these differences were not statistically significant (Fig 1).

**Table 2:** The incidence of IPD among adult patients before and after the introduction of the 13-valent pneumococcal conjugate vaccine (PCV13) in Spain.

Age group (years)	Serotypes	Number of episodes		Incidence*		IPD change from pre-PCV13 to PCV13 (95% CI)	P**
		pre-PCV13	PCV13	pre-PCV13	PCV13		
**18–50**	PCV7	52	19	1.1	0.4	-60.9 (-77.0 to -33.8)	<0.001
	Additional PCV13	155	39	3.3	0.9	-73.0 (-81.0 to -61.7)	<0.001
	non-PCV13	120	73	2.6	1.7	-34.8 (-51.3 to -12.8)	0.004
	All	327	131	7.1	3.0	-57.1 (-65.0 to -47.4)	<0.001
**51–64**	PCV7	38	19	2.5	1.2	-50.2 (-71.3 to -13.7)	<0.001
	Additional PCV13	109	56	7.2	3.6	-48.8 (-62.9 to -29.4)	<0.001
	non-PCV13	76	84	5.0	5.5	10.1 (-19.3 to 50.1)	0.58
	All	223	158	14.7	10.3	-29.5 (-42.5 to -13.5)	<0.001
**65–74**	PCV7	25	10	3.2	1.3	-60.6 (-81.1 to -17.9)	<0.001
	Additional PCV13	74	36	9.3	4.5	-52.0 (-67.8 to -28.6)	<0.001
	non-PCV13	61	79	7.7	9.8	27.7 (-8.6 to 78.4)	0.15
	All	160	125	20.2	15.6	-23.0 (-39.0 to -2.6)	0.03
**≥75**	PCV7	42	24	5.2	2.8	-46.8 (-67.8 to -12.2)	<0.001
	Additional PCV13	102	61	12.6	7.0	-44.4 (-59.5 to -23.6)	<0.001
	non-PCV13	95	110	11.7	12.6	7.74 (-18.1 to 41.8)	0.63
	All	239	195	29.5	22.4	-24.1 (-37.2 to -8.3)	0.005
**All groups**	PCV7	157	72	2.0	1.0	-52.7 (-64.2 to -37.5)	<0.001
	Additional PCV13	440	192	5.7	2.6	-55.0 (-62.0 to -46.7)	<0.001
	non-PCV13	352	345	4.5	4.6	1.0 (-12.9 to 17.2)	0.91
	All	949	609	12.3	8.1	-33.9 (-40.3 to -26.8)	<0.001

*Estimated episodes per 100.000 population (95% CI)

** p-value comparing pre-PCV13 (2008–2009) and PCV13 (2012–2013) periods.

### Serotypes

**Fig 2:**
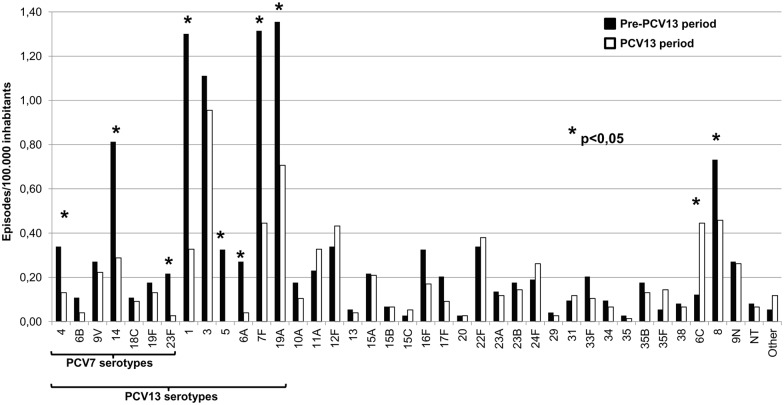
Incidence of invasive pneumococcal disease by serotype and by period. Asterisks indicate serotypes with statistically significant changes (p≤0.05). All p values were ≤ 0.001 with the exception of serotypes 4 (0.012) and 8 (0.049).

The overall IPD due to each of PCV13 additional serotypes (1, 3, 5, 6A, 7F and 19A) significantly declined with the exception of IPD due to serotype 3 which remained stable (Fig 2 and S1 Table). In addition, IPD due to serotypes 4, 14 and 23F, included in PCV7, also showed a statistically significant decrease. No significant changes in the IPD due to non-PCV13 serotypes was observed with the exception of IPD due to serotype 6C, which rose (301.6%; 95%CI, 92.7% to 733.3%), and IPD due to serotype 8, which decreased (-34.9%, 95%CI, -57.1% to -1.2%). This decrease of serotype 8 was only linked to a reduction in the region of Madrid (-53.4%, 95% CI, -74.7% to -14.2%), remaining stable in the other regions (-9.8%, 95% CI, -49.8% to 62.1%).

### Antibiotic susceptibility

Table 3 shows the results of the antibiotic susceptibility to the 8 antimicrobials tested. Percentages of non-susceptibility to penicillin (MIC≥0.12 mg/L, 22.7% vs 26.8%, p = 0.065), cefotaxime (MIC>0.5 mg/L, 10.1% vs 12.5%, p = 0.14), as well as the proportion of MDR isolates (13.1% vs 16.2%, p = 0.08), showed a non-significant increase. Moreover, the proportion of isolates fully resistant to penicillin or cefotaxime (MIC ≥ 2 mg/L) rose throughout the study period (6.4% vs 9.2%, p = 0.043 and 1.5% vs 3.6%, p = 0.006, respectively). Nevertheless, the incidence of IPD due to penicillin-resistant isolates was similar in both periods: 0.79 and 0.75 episodes per 100.000 population, respectively. Similarly, the incidence of IPD caused by those cefotaxime-resistant isolates showed a non-significant increase (62.0%, 95% CI, -17.1% to 216.6%, p = 0.154) from 0.18 to 0.29 episodes per 100.000 population. IPD caused by MDR isolates also remained stable: 1.60 and 1.31 episodes per 100.000 population, respectively (-17.7%, 95% CI, -36.8% to 7.1%, p = 0.14). There were no statistically significant differences for the remaining antimicrobials.

**Table 3:** Changes in antimicrobial susceptibility to eight antimicrobials before and after the PCV13 introduction.

Antimicrobial	pre-PCV13 (2008–2009)			PCV13 (2012–2013)			p*
	S^#^	I	R	S	I	R	
	n (%)	n (%)	n (%)	n (%)	n (%)	n (%)	
Penicillin	734 (77.3)	155 (16.3)	61 (6.4)	446 (73.2)	107 (17.6)	56 (9.2)	0.043
Cefotaxime	852 (89.8)	83 (8.7)	14 (1.5)	533 (87.5)	54 (8.9)	22 (3.6)	0.006
Erythromycin	748 (78.8)	0 (0)	201 (21.2)	463 (76.1)	0 (0)	146 (23.9)	0.219
Clindamycin	770 (81.1)	0 (0)	179 (18.9)	483 (79.3)	0 (0)	126 (20.7)	0.399
Tetracycline	741 (78.1)	74 (7.8)	134 (14.1)	463 (76.1)	38 (6.2)	108 (17.7)	0.350
Chloramphenicol	880 (92.7)	…	69 (7.3)	571 (93.7)	…	38 (6.3)	0.415
Co-trimoxazole	675 (71.1)	54 (5.7)	220 (23.2)	441 (72.4)	21 (3.5)	147 (24.1)	0.607
Levofloxacin	926 (97.6)	…	23 (2.4)	601 (98.7)	…	8 (1.3)	0.147

* p-value comparing resistant isolates between periods. CLSI breakpoints were used.

^#^ S: susceptible; I: intermediate; R: Resistant. Classical CLSI breakpoints for penicillin (oral: susceptible≤0.06mg/L; intermediate 0.12-1mg/L; and resistant ≥2mg/L) and cefotaxime (meningeal: susceptible≤0.5mg/L; intermediate 1mg/L; and resistant ≥2mg/L) were used.

Two PCV13 serotypes 19A (26.9% and 26.5%, respectively) and 14 (25.5% and 26.5%, respectively) were the major serotypes associated with penicillin non-susceptibility in both periods. Regarding erythromycin-resistance, a shift in the most frequent serotypes was found: serotypes 8 (29.4%), 33F (12.9%), 19A and 23A (10.6% both) were the most frequent in the first period and serotypes 6C (25.6%), 11A (16.3%) and 33F (11.6%) in the second period. In this way, serotype 19A was the most frequent among dual-resistant strains (non-susceptibility to penicillin and resistance to erythromycin) in both periods (36.1% vs 37.6%, respectively). Two nonPCV-13 serotypes associated with dual resistance emerged in the second period: 24F and 6C (15.8% both).

### Molecular typing

After the analysis of 852 isolates by PFGE/MLST we were able to identify the major clones related to specific serotypes previously described in Spain and other European countries [3, 14–17]. This analysis allowed us to classify genotypes according to their antimicrobial resistance profile (See S2 Table). When comparing the two periods, major differences were found in the genetic background of five serotypes (3, 6C, 9V, 11A and 19A). The increase in the IPD caused by serotype 6C was related to an expansion of the clone ST386^6C^ in all three regions, which accounted for 14.3% (1 out of 7 studied) of the first period serotype 6C isolates and for 82.6% (19 out of 23 studied) of the second period isolates. For serotype 3, two major clones were identified: ST180 and ST260. A shift in their proportions was observed, ST260 being predominant in the pre-PCV13 period (66.7%, 16 out of 24 studied), and ST180 in the PCV13 (61.4%, 35 out of 57 studied). In the second period, serotype 9V was only detected in Catalonia and was associated with the emergence of a new penicillin-susceptible clone (ST280^9V^). This clone accounted for 64.7% (11 out of 17 studied) of serotype 9V isolates of Catalonia, while in the first period 42.1% of serotype 9V isolates (8 out 19 studied) belonged to the β-lactam-resistant clone and were detected in all three regions. Among serotype 11A, we detected the emergence of ST6521-MDR isolates (double locus variant of ST156) which accounted for 34.8% (8 out of 23) of the studied strains, mainly in Madrid (5 episodes). Finally, among serotype 19A isolates, the major decline of its incidence was due a decrease of the penicillin- and macrolide-susceptible clones. In fact, we could identify an increase of isolates belonging to the MDR ST320 clone (13.7%, 6 out of 44 and 57.1%, 24 out of 42 studied, respectively) in all three regions.

## Discussion

The present study demonstrated a herd protection of the adult population in Spain after the PCV13 introduction for children, even when vaccination is mainly due under a private voluntary basis. Those results highlight the importance of the herd protection in the epidemiology of the IPD when the target of vaccination is the pediatric population.

In Spain, the estimated vaccine coverage for children remains around 60% for most regions, with the exception of the Autonomous Community of Madrid where the vaccine is included in the official vaccination schedule reaching a coverage above 95% [8, 18–19]. A recent pooled analysis showed that the level of herd protection could be associated with the initial vaccine coverage and the accumulated size of the vaccinated group [20]. In agreement with this, the highest adult IPD decrease due to herd protection was observed in the Madrid region which has the highest vaccine coverage. Although other factors could have influenced this higher decrease in the Madrid region, we were not able to identify them.

Our results are similar to what was reported previously from other European countries, from the US and from Israel [10, 21–25] and show a sharp decrease of the IPD only three years after the PCV13 introduction. This IPD reduction was observed in all age groups and was mainly linked to a reduction of the incidence of bacteremic pneumonia whereas the incidence of meningitis remained stable [26]. However, it is remarkable that the mentioned studies included pediatric populations with higher vaccine coverage than our study.

Overall, the incidence of non-PCV13 serotypes remained stable in the last period, with the exception of a decrease in people aged <50 years. Although we have no explanation for this decrease, it could be partially due to the existence of an outbreak of IPD due to Serotype 8-ST63 among young adults (mainly HIV-infected patients) in the region of Madrid over the 2004–2009 period (pre-PCV13); in fact among non-PCV13 serotypes, only serotype 8 IPD decreased [27,28]. As expected, a reduction of incidence was observed for all PCV13 additional serotypes (1, 3, 5, 6A, 7F and 19A) with the exception of serotype 3, which remained stable (-11.3%, 95%CI -35.0% to 21.1%, p = 0.477). The impact of the PCV13 on the incidence of serotype 3 is a controversial issue as there are different findings. For instance, a reduction of IPD due to serotype 3 has been recently reported in England and Wales by Waight [25], whereas no significant changes have been reported in the US and other European Countries [10, 17, 22, 24]. This serotype is rarely found colonizing children [29,30] whereas it is one of the first causes of IPD in adults as well as the fact that it is also associated with severe disease and high mortality rates [31, 32]. In this way, we detected that serotype 3 was the leading cause of IPD in the PCV13 period (12%) whilst it was only the fourth (9.1%) of the pre-PCV13 period (after serotypes 7F, 19A and 1). Probably, higher vaccine coverage and a longer vaccination period are needed to observe herd protection on the adult population [20]. However, the expansion of the PCV13 vaccination to the adult population at risk could be more successful to reduce the serotype 3 disease in adults. On the other hand, a change in their genetic background was observed when the local serotype 3 clone (ST260) has been partially replaced by the widely disseminated serotype 3 clone (ST180). This shift could not be explained by antimicrobial pressure (both STs are fully antimicrobial susceptible) and probably reflects clonal fluctuations of serotypes as previously described.

Regarding serotype 19A, despite the observed reduction of the IPD due to this serotype, the number of isolates belonging to ST320^19A^ increased over the study period, which is a matter of concern since isolates of this clonal complex show a MDR pattern. These results are in agreement with those published in the USA from the late PCV7 period and the early PCV13 [5,7, 33–35] but, as data of molecular epidemiology after PCV13 introduction in Europe is scarce, we could not compare the occurrence of this phenomenon in other countries. Moreover, a reduction of isolates belonging to ST320^19A^ has also been detected in a pediatric population from the USA [35] after the PCV13 introduction, even though this reduction was lower than that observed for the susceptible clones. In fact, it seems that the persistence of the ST320^19A^ over the remaining 19A clones may be due to a combination of factors including an improved colonizing ability [36], a change in their metabolic profile [37] and an enhanced resistance to the antimicrobial stress due its MDR pattern.

Regarding the non-vaccine serotypes, their incidence remained stable for most of them with the exception of serotype 6C and the previously described serotype 8. In fact, serotype 6C was the only non-vaccine serotype that significantly increased over the study period, related to an expansion of the CC386^6C^. This multidrug resistant clone was identified as an emerging lineage in Spain in 2009 [38] and a rise in its frequency has also been detected in the UK [39] and France [16] after the PCV7 introduction and in the early PCV13 period. These data could suggest a clonal replacement of those vaccine serotypes included in the serogroup 6 (6A and 6B). Nevertheless, it is remarkable that cross-reactivity between serotypes 6A and 6C has been reported [40] and, therefore, the PCV13 introduction should theoretically protect against serotype 6C colonization and reduce their impact on the adult IPD, as observed by some authors [10,41]. Our results could suggest a minor effect of the cross-reactivity between these serotypes but also the need for a higher coverage to observe this impact. Moreover, several works that were unable to detect a reduction of 6C isolates after the PCV13 introduction have been published [24, 25, 42]. In any case, the expansion of this clone showing non-susceptibility to penicillin, and macrolides and tetracycline resistance should be monitored.

Data regarding antimicrobial resistance after the PCV13 introduction is still scarce [10, 17, 23] but a decrease in the prevalence of penicillin- and cefotaxime-resistance has generally been reported. In our study, although we have observed an increase in the proportion of non-susceptibility to penicillin, cefotaxime, and MDR isolates, the incidence of IPD due to these isolates remained stable. These results were related to a drastic reduction of the IPD caused by those prevalent susceptible clones (mainly ST306^1^, ST289^5^, ST392^17F^ and ST1201^19A^), to a stabilization of the IPD caused by some multidrug resistant clones (ST230^24F^, ST320^19A^) and to an emergence of new resistant clones (ST6521^11A^ and ST386^6C^). Of note, ST6521^11A^ is a recombinant clone previously associated to serotypes 14 and 9V (included in the PCV13) and it was mainly detected in Madrid, the region with higher coverage. This clone has been shown to have the ability to evade the immune system [43]. On the other hand, the emergence of ST386^6C^ clone was detected in all three regions, even in Madrid. Then, probably, the cross-reactivity of 6A with 6C needs a longer period as occurred for 6B and 6A [38].

The main strength of our study is the valuable information that it gives through a well characterized population. Since the impact of the conjugate vaccines depends on, obviously, the pre-existing proportion of serotypes of the target population, but also depends on the circulating clones, it is mandatory to study the pneumococcal population from a global perspective of serotype-genotype. However, other factors could have contributed to the observed changes in IPD such as variations in the overall population at risk of suffering IPD, which is the main limitation of our study. In the same line, the selection of resistant isolates for molecular typing in the pre-PCV13 period could have hidden the emergence or spread of antibiotic susceptible clones. However, the clonal composition of Spanish invasive pneumococci in the prePCV13 period has been previously analyzed. Other limitations of this study are the lack of information about the 23-valent pneumococcal polysaccharide vaccination (with coverage nearly 40% in adults over 65), the immune status of the patients and the existence of comorbidities, whose changes could have affected the incidence of IPD [44]. Finally, since up to 70% of the strains were from Catalonia, this could be a bias of our study.

## Conclusions

The decrease in the incidence of IPD observed in the period 2012–2013 was due to a decrease in the incidence of PCV13 serotypes which demonstrates the importance of the herd immunity. In spite of that, after the PCV13 introduction new clones have appeared (ST386^6C^, ST6521^11A^) and others have increased despite being clones related to serotypes included in the vaccine (ST180^3^ and ST320^19A^). In addition, in our geographical area, the rates of resistance to penicillin and cefotaxime have increased in association to an expansion of MDR clones. On account of all this, the surveillance of the pneumococcal invasive disease continues being necessary as well as the development of broader conjugate vaccines.

## Supporting information

S1 Table Change of adult IPD incidence by serotype from pre-PCV13 to PCV13. (PDF)

S2 Table Results of molecular typing. (XLSX)

S3 Table Demographic characteristics of the study population by hospital. (XLSX)

S1 Fig Hospital’s location, demographics and number of episodes by period. (TIF)
