# Supplementary material for: Decrease of invasive pneumococcal disease (IPD) in adults after introduction of pneumococcal 13-valent conjugate vaccine in Spain
Source: PLoS One. 2017 Apr 6;12(4):e0175224. doi: 10.1371/journal.pone.0175224 (PMC5383258; doi:10.1371/journal.pone.0175224)
Supplement: S1 Table — (PDF) [file pone.0175224.s001.pdf]

S1 Table. IPD change from pre-PCV13 to PCV13 period.

Serotypes			Estimated number of episodes		Incidence*		IPD change from pre-PCV13 to PCV13 (95% CI)	p-value**
			pre-PCV13	PCV13	pre-PCV13	PCV13		
PCV7	PCV13	4	26	10	0,34	0,14	-60.36 (-80.88 to -17.83)	0.012
		6B	8	3	0,11	0,04	-61.35 (-89.75 to 45.77)	0.227
		9V	21	17	0,27	0,23	-16.60 (-55.99 to 58.23)	0.628
		14	63	22	0,81	0,30	-64.00 (-77.85 to -41.52)	<0.001
		18C	8	7	0,11	0,09	-9.83 (-67.30 to 148.76)	1
		19F	14	10	0,18	0,14	-26.36 (-67.30 to 65.84)	0.542
		23F	17	2	0,22	0,03	-87.87 (-97.20 to -47.51)	0.001
		1	101	25	1,30	0,34	-74.49 (-83.53 to -60.47)	<0.001
		3	86	74	1,11	0,99	-11.35 (-35.02 to 21.07)	0.477
		5	25	0	0,33	0,00	-95.88 (-99.44 to -69.58)	<0.001
		6A	21	3	0,27	0,04	-85.28 (-95.61 to -50.64)	<0.001
		7F	102	35	1,31	0,46	-64.64 (-75.91 to -48.08)	<0.001
		19A	105	55	1,36	0,73	-46.00 (-61.04 to -25.21)	<0.001
non-vaccine serotypes		6C	9	35	0,12	0,46	301.61 (92.68 to 733.33)	<0.001
		8	57	36	0,73	0,47	-34.90 (-57.12 to -1.19)	0.049
		10A	14	8	0,18	0,11	-41.11 (-75.29 to 40.45)	0.287
		11A	18	25	0,23	0,34	43.06 (-21.88 to 162.47)	0.286
		12F	26	34	0,34	0,45	34.77 (-19.09 to 124.72)	0.302
		13	4	3	0,05	0,04	-22.7 (-82.7 to 244.8)	1
		15A	17	16	0,22	0,22	-3.01 (-50.98 to 91.94)	1
		15B	5	5	0,07	0,07	3.1 (-70.2 to 255.9)	1
		15C	2	4	0,03	0,05	106.2 (-62.2 to 1023.6)	0,446
		16F	25	13	0,33	0,18	-46.41 (-72.58 to 4.71)	0.075
		17F	16	7	0,20	0,09	-54.91 (-81.45 to 9.65)	0.094
		20	2	2	0,03	0,03	3.1 (-85.5 to 629.9)	1
		22F	26	29	0,34	0,39	18.91 (-29.68 to 101.21)	0.593
		23A	10	9	0,14	0,12	-15.68 (-65.06 to 103.67)	0.824
		23B	14	11	0,18	0,15	-19.03 (-63.24 to 78.25)	0.691
		24F	15	20	0,19	0,27	37.36 (-29.63 to 168.10)	0.400
		29	3	2	0,04	0,03	-31.3 (-88.5 to 311.5)	1
		31	7	9	0,09	0,12	32.45 (-50.64 to 255.87)	0.624
		33F	16	8	0,20	0,11	-48.48 (-77.94 to 20.48)	0.153
		34	7	5	0,09	0,07	-26.36 (-76.64 to 132.02)	0.775
		35	2	1	0,03	0,01	-48.5 (-95.3 to 468.2)	1
		35B	14	10	0,18	0,14	-26.36 (-67.30 to 65.84)	0.542
		35F	4	11	0,05	0,15	183.29 (-9.75 to 792.86)	0.073
		38	6	5	0,08	0,07	-14.09 (-73.79 to 181.69)	1
		9N	21	20	0,27	0,27	-1.86 (-46.78 to 81.16)	1
		NT	6	5	0,08	0,07	-14.1 (-73.8 to 181.7)	1
		Other	4	9	0,08	0,12	54.6 (-45.0 to 334.4)	0.565

* Episodes per 100.000 population

**p-value comparing pre-PCV13 (2008-2009) and PCV13 (2012-2013) periods
