# Supplementary figures and images for: Decrease of invasive pneumococcal disease (IPD) in adults after introduction of pneumococcal 13-valent conjugate vaccine in Spain
Source: PLoS One. 2017 Apr 6;12(4):e0175224. doi: 10.1371/journal.pone.0175224 (PMC5383258; doi:10.1371/journal.pone.0175224)

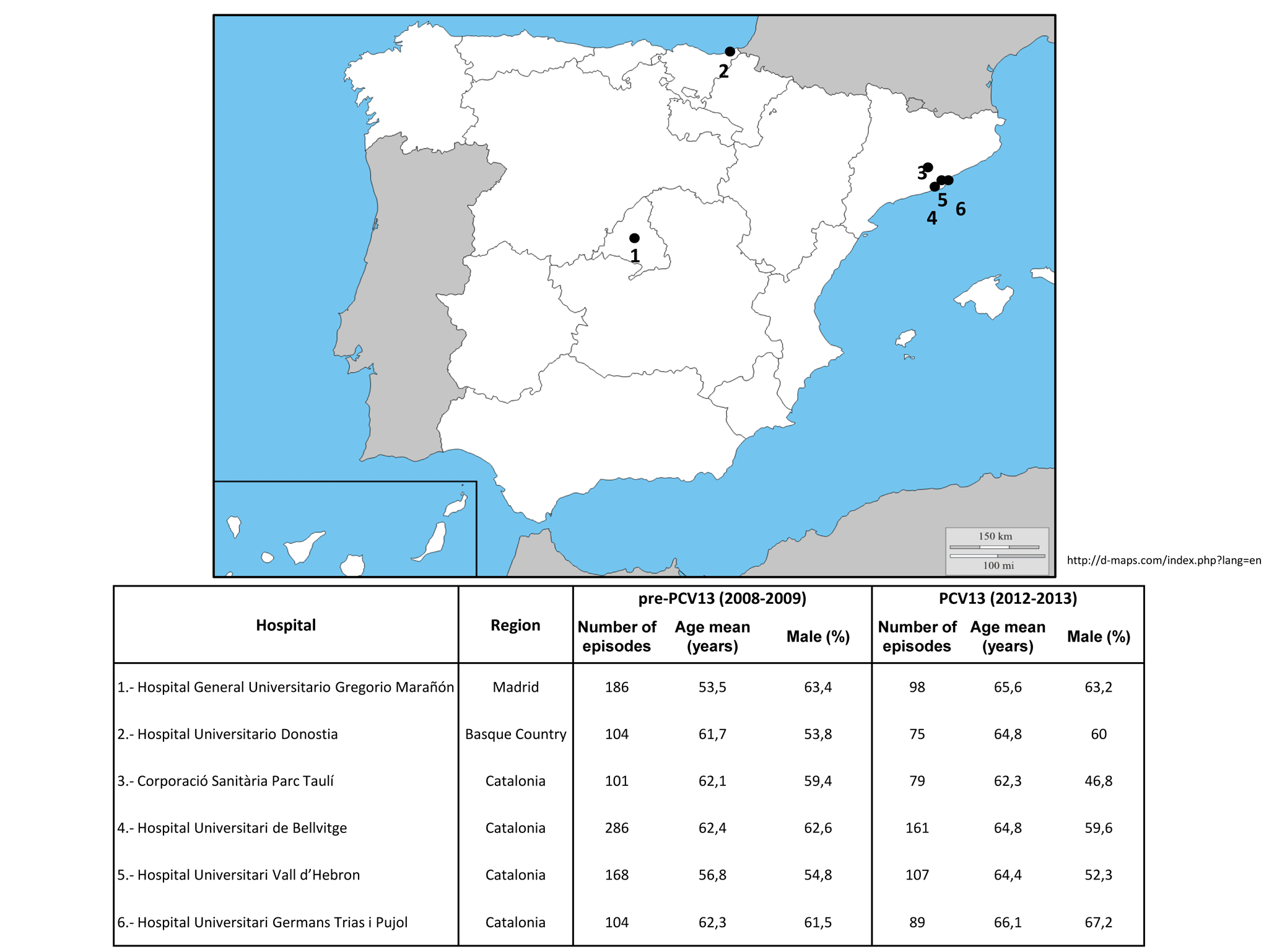

Supplement: S1 Fig — (TIF) [file pone.0175224.s004.tif]
